# The Prevalence of Dyslipidemia and Hyperglycemia among Stroke Patients: Preliminary Findings

**DOI:** 10.1155/2019/8194960

**Published:** 2019-10-30

**Authors:** Iyad Ali, Mahmoud Abuissa, Anan Alawneh, Omar Subeh, Ahmad Abu Sneineh, Sabreen Mousa, Israa' Deeb, Hiba Rayyan

**Affiliations:** ^1^Department of Biochemistry and Genetics, Faculty of Medicine and Health Sciences, An-Najah National University, Nablus, State of Palestine; ^2^Department of Internal Medicine, Al-Watani Governmental Hospital, Nablus, State of Palestine; ^3^Department of Medicine, Faculty of Medicine and Health Sciences, An-Najah National University, Nablus, State of Palestine; ^4^Department of Pharmacy, Faculty of Medicine and Health Sciences, An-Najah National University, Nablus, State of Palestine

## Abstract

**Background/Aim:**

Stroke or cerebrovascular accident is defined as sudden or sub acute onset of focal neurologic deficit, caused by the interruption of blood flow to parts of the brain. In this study, we aimed to investigate the prevalence of dyslipidemia and hyperglycemia among stroke patients in Palestine.

**Materials and Methods:**

A total of 70 patients with stroke were included in a cross-sectional study between November 2017 and February 2018. Stroke patients were diagnosed based on a CT scan reviewed by a neurologist. Fasting venous blood samples were collected to measure the lipid profile (cholesterol, low-density lipoproteins (LDL), triacylglycerol (TAG), high-density lipoproteins (HDL)), fasting blood glucose (FBG), and glycosylated hemoglobin (Hb_A1c_) levels. An interview-based questionnaire, included background data, past medical history, family history, and other risk factors for stroke, was filled for each patient.

**Results:**

Based on our results, 28.57% of patients had high LDL, 17.1% had high cholesterol, 15.7% had high TAG and 61.3% had low HDL. About half of the patients (51.4%) had abnormal Hb_A1c_ and abnormal FBG (52.8%). The majority (67.1%) of patients were males, whereas, 11% of patients were obese (BMI of more than 30 kg/m^2^) and 51.4% were smokers. Regarding the family history of diseases, 81% of patients had a family history of hypertension, 50% had a family history of stroke, and 58% had a family history of diabetes mellitus.

**Conclusion:**

Male gender and smoking were most likely to increase the risk of stroke. Risk factors like low HDL, high LDL, high FBG, high Hb_A1c_, and hypertension contribute substantially to the incidence of stroke. A family history of stroke, hypertension and diabetes were significant risk factors for stroke.

## 1. Introduction

Stroke, cerebrovascular accident (CVA), is one of the leading causes of morbidity and mortality worldwide. In fact, it is the second leading cause of death worldwide, responsible for 165,000 deaths occuring each year in United States Alone [1]. In Palestine, there are a quite a large number of CVA patients according to a study done in northern Palestine, which showed that the annual crude incidence rate of stroke was 51.4 per 100,000 persons [2]. Stroke is caused by the interruption of the blood supply to the brain, this cuts off the supply of oxygen and nutrients, causing damage and ischemia to the brain tissues [3, 4]. The most common symptom of stroke is sudden weakness or numbness of the face, arm or leg, most often on one side of the body which is considered a focal neurological deficit [5]. Other symptoms include: confusion, difficulty speaking or understanding speech, difficulty seeing with one or both eyes, difficulty walking, dizziness, loss of balance or coordination, severe headache with no known cause, and fainting or unconsciousness [6]. Lifestyle habits as tobacco and alcohol use, sedentary life and obesity, high blood pressure, atrial fibrillation, hyperlipidemia, diabetes, and atherosclerosis can be considered as controllable risk factors. On the other hand, uncontrollable risk factors for stroke include age, gender, race, family history and previous history of stroke, or transient ischemic attack [6].

Dyslipidemia and high cholesterol levels are risk factors for many diseases, including hypertension, stroke, ischemic heart disease, and peripheral vascular disease [7]. They are defined as disorders of lipoprotein metabolism, including lipoprotein overproduction or deficiency [8, 9]. Elevated LDL levels appear to increase the risk of ischemic stroke, while low HDL levels appear to be associated with a greater risk, whereas the importance of high TAG levels is less clear [8, 10]. The discordant results of observational studies might result from the heterogeneity of stroke, since dyslipidemia is less likely to play a major role in the pathogenesis of some ischemic stroke subtypes (e.g., lacunar and cardio-embolic strokes) [9]. Some epidemiological studies have provided conflicting findings regarding the association of dyslipidemia with ischemic stroke, while elevated LDL levels might increase the risk of hemorrhagic stroke [11, 12]. In clinical trials, statins consistently reduced the risk of ischemic stroke in patients with or without coronary artery disease, whereas the data on the effects of other lipid modifying drugs on stroke risk are limited. In patients with a previous stroke, statins reduce the risk of both ischemic stroke and other vascular events, but also increase the risk of hemorrhagic stroke [13–15].

Another factor associated with stroke admission is hyperglycemia. Several studies showed that diabetes is a central risk factor for ischemic stroke [16, 17]. Meta-analysis done by Capes et al., found the higher risk of death after ischemic stroke in patients with higher blood glucose levels [18]. But the relationship between blood glucose levels and risk of stroke is less certain than the strong relation between diabetes and stroke [18]. Two-thirds of patients with post stroke hyperglycemia had either impaired glucose tolerance or diabetes mellitus at 12 weeks' post-stroke [19]. It is indicated by many experimental and animal studies that hyperglycemia predicts higher stroke mortality independent of stroke type, severity of stroke or age. The results suggest that hyperglycemia may be directly associated to poor outcomes by exacerbating acute brain injury [20, 21]. Berger and Hakim suggested that hyperglycemic patients develop more pronounced cerebral edema and worsening clinical outcome after having stroke [22]. The severity of acute stroke is associated with the incidence and degree of hyperglycemia and the mortality was significantly increased in hyperglycemic patients [23].

Undoubtedly, dyslipidemia and diabetes are two of the common disorders all over the world, and they are considered as risk factors for many diseases, while stroke is a clinical condition that directly and badly affects life and may result in death. Therefore, the aim of this study was to determine the prevalence of dyslipidemia and hyperglycemia in stroke patients and to assess the risk factors associated with stroke among these patients and this would influence the treatment course and prognosis of stroke per se.

## 2. Materials and Methods

This is a cross-sectional study conducted at Al-Watani governmental hospital in Nablus district of Palestine, in the period between the 1^st^ of November 2017 and the 1^st^ of March 2018. The study population consisted of all stroke patients admitted to Al-Watani hospital with a confirmed CT scan. Patients without a confirmed CT scan, those suspected of having a transient ischemic attack or patients who refused to undergo the interview or give blood sample, were excluded. The sample size of the study included all patients who were admitted with a stroke during the period of 4 months and met the inclusion criteria. During this period, 90 patients were admitted as stroke patients; out of them 20 patients were excluded for not meeting the inclusion criteria. This study was conducted after obtaining an approval from the institutional review board and at An-Najah National University and from the Palestinian Ministry of Health (MOH). A verbal consent was obtained from each patient before including him/her in the study. Approval for blood sample withdrawal was obtained from the MOH and from each patient. For each patient who was included in this study, background data (Age, gender, and social status), past medical history (hypertension, diabetes, atrial fibrillation, ischemic heart disease, transient ischemic attack, and previous history of stroke), other risk factors (smoking, alcohol use, and obesity/BMI) and family history were obtained.

Fasting venous blood samples were collected from stroke patients to measure cholesterol, TAG, LDL, HDL, FBG, and Hb_A1c_. Other laboratory data, included C-reactive protein (CRP), creatine kinase (CK), liver enzymes (ALT, AST), Kidney function test (serum creatinine, serum urea and blood urea nitrogen (BUN)), blood cells count (white blood cells (WBC), platelets and hemoglobin (Hb)), and electrolytes (potassium, sodium and calcium), were obtained from patients' files. Hyperlipidemia was defined as cholesterol equal or more than 200 mg/dl, TAG of equal or more than 200 mg/dl, LDL of equal or more than 130 mg/dl, and HDL of less than 40 mg/dl in men and less than 50 mg/dl in women [24]. Patients were diagnosed as diabetic if FBG level is equal or more than 126 mg/dl on more than one occasion or random blood glucose level more than 200 mg/dl on one occasion with symptoms of hyperglycemia or Hb_A1c_ equal or more than 6.5% [25]. Patients who were normoglycemic at the time of presentation, but with a history of diabetes, taking insulin or oral hypoglycemic were also labelled as diabetics. A smoker was defined as a person who smoked at least one cigarette per day for the preceding 3 months or more, or use tobacco in any form [26]. Obesity was defined as a person with a BMI of 30 kg/m^2^ or more. We analyzed our data using Statistical Package for Social Sciences version 21 (SPSS Inc., Chicago, IL, USA).

## 3. Results

During the study period, a total of 90 stroke patients were admitted to Al-Watani governmental hospital, out of them, 70 patients were participated in this cross-sectional study, giving a response rate of 77%. The patients had a mean age of 68.7 years with 78.5% of them aged above 60 years, and 61.5% of them were males. The demographic distribution of age and gender in the study is shown in Table 1.

Studying the co morbidity, 53 (75.7%) patients had hypertension, 34 (49%) patients had diabetes mellitus, and 8 (11%) patients had transient ischemic attack. Thirty-six patients (51%) were smokers, the majority of the smokers were males, and 8 (11%) patients were obese with a BMI above 30 kg/m^2^ with a mean of 31.6 kg/m^2^. The co morbidities' incidence rates are demonstrated in Table 2.

Table 3 shows that 57 (81%) patients had a family history of hypertension, 35 (50%) patients had a family history of stroke and 41 (58%) patients had a family history of diabetes mellitus (Table 3).

Regarding the lipid profile and hyperglycemia, it was found that, 20 patients (28%) had LDL greater than 130 mg/dl, 12 patients (17%) had cholesterol equal or greater than 200 mg/dl, 11 (15.7%) patients had TAG equal or greater than 200 mg/dl, 43 (61.3%) patients had low HDL (Table 4). Thirty six patients (51.4%) had Hb_A1c_ levels equal or greater than 6.5% and 37 patients (52.9%) had FBG equal or greater than 126 mg/dl.

The mean and standard deviation of the main variables in this study are shown in Table 5. The mean age of the patients was 68.7 years, the main BMI was 27.6 kg/m^2^. The mean level of cholesterol, LDL, HDL and TAG were 163.1, 114.9, 42.3, and 133.8 mg/dl, respectively. The mean of the HbA_1c_ was 6.8%, while the mean of FBG was 158 mg/dl.

## 4. Discussion

Stroke is classically characterized as a neurological deficit attributed to an acute focal injury of the central nervous system by a vascular cause, including cerebral infarction, intracerebral hemorrhage, and subarachnoid hemorrhage, and is a major cause of disability and death worldwide [27]. The presentation of stroke is variable, ranging from subtle to severe, depending on the area of brain involved and the nature of the attack [28]. The role of dyslipidemia in the pathogenesis of stroke is less clear. Studies have shown conflicting findings regarding the association between dyslipidemia and stroke [11].

In this 4 months' hospital-based study, the prevalence of dyslipidemia, hyperglycemia, and other stroke risk factors were studied among stroke patients. The male to female ratio was almost 3 : 2, similar to other studies [29, 30]. The higher incidence of stroke among male may be attributed to high prevalence of smoking among Palestinian men and the consumption of more fatty food. In addition, the hormonal effects of estrogen also have a protective effect against stroke in females. Although a study from Oxford shire, showed that males are more affected than females by genetic factors, the family history is more likely to be found in females than in males [31]. Regarding the age distribution of stroke patients in the study, the mean age was 68.7 years (±10.7) which is similar to a result of a study in India and a study in Palestine 10 years ago that showed a mean age of 69 years [2, 30]. The majority, 55 (78.5%) patients, were above the age of 60 years. Again this result is almost similar to the previous study conducted in Palestinian that found 82% of patients were above the age of 60 years [2]. These results indicate that the incidence of stroke is higher for those who are above 60 years old. On the other hand, the mean BMI of stroke patients was 27.6 kg/m^2^ (±2.5) with 8 (11%) patients above 30 kg/m^2^, and this is higher than a study carried out in Japan [32]. Although obesity and higher BMI is established as a risk factor for coronary artery disease, its role as a risk factor for stroke remains controversial.

Although there are many possible causes of human disease, family history is often one of the strongest risk factors for common disease complexes such as stroke, cancer, and diabetes. We found that a family history for HTN, stroke, or DM was associated with the increased incidence of stroke. Family history of HTN (81%) was found as the main cerebrovascular risk factor in stroke, followed by a family history of DM (58%) and stroke (50%). Therefore, family history is thought to be a good predictor of stroke risk because family members most closely represent the unique genomic and environmental interactions that an individual experiences [33].

The involvement of hypertension, diabetes, cigarette smoking, and others in the formation of stroke is widely established [19, 34, 35]. Hypertension (75.7%) was found to be the main stroke risk factor, followed by smoking (51%) and diabetes (49%). Moreover, the high incidence of hypertension increases the risk of stroke. Although our understanding of the benefits of treating high blood pressure, diabetes, and smoking for the secondary prevention of strokes is evolving, we have identified a significant need for improvement in overcoming these risk factors. Stroke prevention clinics may need to be more actively involved in the management of these modifiable risk factors if we are to significantly impact the risk of recurrent stroke [36].

The lipid profile of stroke patients was studied and it was found that there were 12 (17%) patients with cholesterol level ≥ 200 mg/dl and the mean total cholesterol was 163.1 mg/dl (±43.3), in agreement with other results which showed no significant correlation between cholesterol level and the risk of stroke [14, 37].Other studies showed an increased risk of stroke in patients with higher levels of cholesterol [38, 39]. This may indicate that the role of high cholesterol levels as a risk factor for stroke is still unclear. High level of TAG (>200 mg/dl) were found in 11 patients (15.7%), and the mean TAG level was 133.8 mg/dl (±61.1). These results are similar to several studies that showed the TAG level ranging from 127 to 154 mg/dl among stroke patients [40]. These observations may indicate that the relationship between elevated TAG levels and the risk of stroke is still lacking, and this is in agreement with previous studies showed that no clear relationship between elevated TAG levels and risk of stroke [9, 40]. The mean LDL level was 114.9 mg/dl (±33.4), 20 patients had LDL above 130 mg/dl (28.57%). Conflicting results are reported in the literature about the relationship between elevated levels of LDL and risk of stroke [8, 41]. Among male patients, 23 (32.8%) of them had HDL level less than 40 mg/dl, while, 20 (28.5%) female patients had HDL less than 50 mg/dl, and the mean HDL level for both males and females was 42.3 mg/dl (±15.3). Several studies showed similar findings and suggested that lower levels of HDL are associated with increased risk of stroke, while high levels of HDL are considered as a slight protective indicator against stroke [14, 42]. On the other hand, a study conducted in Hawaii, showed no clear relationship between low levels of HDL and the risk of having stroke [43].

The results of Hb_A1c_ showed that 36 patients (51.4%) had Hb_A1c_ ≥ 6.5% with a mean of 6.8% (±1.6). The FBG ≥ 126 mg/dl was found in 37 (52.9%) patients with a mean of 158 mg/dl (±74.3). The results about Hb_A1c _and FBG were similar to other studies that showed a relationship between hyperglycemia and high Hb_A1c_, and the risk of developing stroke [16–18]. In diabetic patients, several mechanisms suggest that the prolonged hyperglycemia leads to stroke. These include vascular endothelial dysfunction, increased early-age arterial stiffness, systemic inflammation and thickening of the capillary basal membrane [44].

A limitation of the study is the lack of the different stroke subtypes, mainly in the subgroup of patients with lacunar infarcts. Lacunar infarcts are the ischemic stroke subtype with a better functional prognosis. Within that subgroup the main comorbidities are HTA and diabetes [34]. The small sample size is a major limitation of such studies, therefore, a cohort study involving more patients may produce stronger evidence about the effect of dyslipidemia and hyperglycemia on the occurrence of stroke.

## 5. Conclusions

Most of the patients with stroke had low HDL levels, high levels of FBG and Hb_A1c_. Hypertension, DM, smoking and family history of HTN and DM are significant risk factors for the incidence of stroke. Less patients had high LDL, high cholesterol and high TAG, making the effect of these parameters on the incidence of stroke is still controversial.

## Figures and Tables

**Table 1:** Demographic distribution of age and gender.

Gender and age	Number of cases	Percentage
Male	43	61.5
Female	27	38.5
Age over 60 years	55	78.5
Age under 60 years	15	21.5

**Table 2:** Co morbidities associated with stroke.

Co morbidities	Number of cases	Percentage
HTN	53	75.7
DM	34	49
TIA	8	11
Smoker	36	51
BMI > 30	8	11

BMI: body mass index, TIA: transient ischemic attack, DM: diabetes mellitus, HTN: hypertension.

**Table 3:** Family history distribution.

Familial history	Number of cases	Percentage
HTN	57	81
Stroke	35	50
DM	41	58

DM: diabetes mellitus, HTN: hypertension.

**Table 4:** Lipid profile and fasting blood glucose and Glycosylated Hemoglobin levels in stroke patients.

Test	Number of cases	Percentage
LDL ≥ 130 mg/dl	20	28
Cholesterol ≥ 200 mg/dl	12	17
Triglyceride ≥ 200 mg/dl	11	15.7
Low levels of HDL	23 males	32.8
	20 females	28.5
Hb_A1c_ ≥ 6.5%	36	51.4
FBG ≥ 126 mg/dl	37	52.9

FBG: fasting blood glucose, Hb_A1c_: glycosylated hemoglobin.

**Table 5:** Mean and standard deviation of age, BMI, lipids, glycosylated hemoglobin and fasting blood sugar.

Parameter	Mean (±SD)
Age (years)	68.7 (±10.7)
Obesity (BMI) (kg/m^2^)	27.6 (±2.5)
Cholesterol (mg/dl)	163.1 (±43.3)
LDL (mg/dl)	114.9 (±33.4)
HDL (mg/dl)	42.3 (±15.3)
Triglyceride (mg/dl)	133.8 (±61.1)
HbA1c (%)	6.8 (±1.6)
FBG (mg/dl)	158 (±74.3)

BMI: body mass index, FBG: fasting blood glucose, Hb_A1c_: glycosylated hemoglobin.

## Data Availability

All data will be available on request.
